# Incidence and Risk Factors for Incident Syphilis among HIV-1-Infected Men Who Have Sex with Men in a Large Urban HIV Clinic in Tokyo, 2008−2015

**DOI:** 10.1371/journal.pone.0168642

**Published:** 2016-12-16

**Authors:** Takeshi Nishijima, Katsuji Teruya, Satoshi Shibata, Yasuaki Yanagawa, Taiichiro Kobayashi, Daisuke Mizushima, Takahiro Aoki, Ei Kinai, Hirohisa Yazaki, Kunihisa Tsukada, Ikumi Genka, Yoshimi Kikuchi, Shinichi Oka, Hiroyuki Gatanaga

**Affiliations:** AIDS Clinical Center, National Center for Global Health and Medicine, Tokyo, Japan; University of Malaya, MALAYSIA

## Abstract

**Background:**

The epidemiology of incident syphilis infection among HIV-1-infected men who have sex with men (MSM) largely remains unknown.

**Methods:**

The incidence and risk factors for incident syphilis (positive TPHA and RPR> = 1:8) among HIV-1-infected MSM who visited a large HIV clinic in Tokyo for the first time between 2008 and 2013 were determined, using clinical data and stored blood samples taken every three months for screening and determination of the date of incident syphilis. Poisson regression compared the incidence of syphilis at different observation periods.

**Results:**

Of 885 HIV-1-infected MSM with baseline data, 34% either presented with active syphilis at baseline (21%) or became infected with syphilis during follow-up (13%). After excluding 214 patients (MSM with syphilis at baseline (n = 190) and no follow-up syphilis test (n = 24)), of 671 men, 112 (17%) developed incident syphilis with an incidence of 43.7/1,000 person-years [95% CI, 36.5–52.3]. The incidence decreased slightly during observation period although the trend was not significant (2008–2009: 48.2/1,000 person-years, 2010–2011: 51.1/1,000 person-years, 2012–2013: 42.6/1,000 person-years, 2014 to 2015: 37.9/1,000 person-years, p = 0.315). Multivariable analysis identified young age (<33 years versus >40, HR 4.0, 95%CI 2.22–7.18, p<0.001), history of syphilis at baseline (HR 3.0, 95%CI 2.03–4.47, p<0.001), positive anti-amoeba antibody (HR 1.8, 95%CI 1.17–2.68, p = 0.006), and high baseline CD4 count (CD4 ≥350 /μL versus CD4 <200, HR 1.6, 95%CI 1.00–2.53, p = 0.050) as risk factors for incident syphilis. Incidence of syphilis was particularly high among young patients (age <33 years: 60.1/1,000 person-years). Interestingly, 37% of patients with incident syphilis were asymptomatic.

**Conclusions:**

Although incidence of syphilis did not increase during the observation period, it was high among HIV-1-infected MSM, especially among young HIV-1-infected MSM and those with history of syphilis, in Tokyo. Regular screening for syphilis needs to be strictly applied to this population.

## Introduction

Syphilis is a curable sexually transmitted infection caused by *Treponema pallidum*. However, recently there had been resurgence in reported syphilis cases in resource-rich settings, with the majority of cases being men who have sex with men (MSM), particularly those with HIV-1 infection [1,2]. The Japanese law required physicians to report new cases of both HIV-1 infection and syphilis. Although the reported number of new HIV-1-infected cases, mostly comprised of Japanese MSM, has plateaued with approximately 1,500 per year since 2007 [3], the reported number of syphilis cases has been rapidly increasing from 828 in 2010 to 1671 in 2014 and to 2698 (provisional value) in 2015 [4]. Most of the reported cases were from urban city areas such as Tokyo, Osaka, and Nagoya. Among 1226 reported syphilis cases in 2013, 81% were men. Among these men, 52% reported homosexual contact as the route of transmission. This suggested that a substantial proportion of syphilis epidemic in Japan was attributed to syphilis infection among MSM [5].

However, this report did not stratify new syphilis cases by HIV-1 infection status. The incidence of syphilis among HIV-1 infected MSM in Japan was still unknown. Therefore, we used the data of the patients attending the AIDS Clinical Center in Tokyo, the largest clinic for HIV care in Japan with most patients being MSM [6], to determine the incidence and risk factors of incident syphilis infection among HIV-1-infected MSM, using clinical data and stored blood samples taken every three months to screen all patients and to determine the date of incident syphilis, attempting to present epidemiological data of syphilis epidemic in this population during 2008−2015 in Japan.

## Methods

### Ethics Statement

This study was approved by the Human Research Ethics Committee of the National Center for Global Health and Medicine (NCGM), Tokyo, Japan (G-002009-00). The study protocol did not require written informed consent since the stud**y** only used data of anonymized patients obtained from a routine practice and from stored serum samples, which were obtained with written informed consent [7]. The study was conducted according to the principles expressed in the Declaration of Helsinki.

### Study setting

The AIDS Clinical Center, NCGM, Tokyo, was established in 1997 and had accumulatively registered approximately 4000 patients [6]. Considering that the total reported number of patients with HIV-1 infection in Japan is 27,413 by the end of 2015, this clinic treats approximately 15% of the HIV-1 infected patients in Japan [3].

### Study subjects

The study population was HIV-1-infected MSM who visited our clinic for the first time between January 2008 and December 2013. The following exclusion criteria were applied; 1) patients who visited the clinic for a second opinion or those who were referred to other facilities on their first/second visit, because some baseline data were likely missing and the follow-up period was short in these patients, 2) patients younger than 20 years of age, because the Ethical Guidelines for Medical and Human Research Involving Human Subjects published by the Japanese Ministry of Health, Labour and Welfare require a written consent by parents/guardians for patients under age of 20 and it is impractical to obtain such consent for this study. Based on the results of baseline data analysis, we also excluded the following patients; 3) patients who had a confirmed active syphilis at baseline based on serum Rapid Plasma Reagin (RPR) titer ≥1:8 and positive *Treponema pallidum* Latex Hemagglutination Assay (TPHA) [8], and 4) patients who lacked follow-up syphilis test. At our clinic, the follow-up syphilis tests were generally performed at least every year and at the discretion of the treating physician. The study patients were followed up until December 31, 2015.

### Measurements and definitions

Serum Rapid Plasma Reagin test (RPR) [“Sankoh” (EIDIA Co, Tokyo)], *Treponema pallidum* latex hemagglutination assay (TPHA), CD4 cell count (categorized into 3 groups: CD4 count <200 /μL, 200–349 /μL, and ≥350 /μL), HIV-1 viral load, hepatitis C antibody (HCVAb), hepatitis B surface antigen (HBsAg), core antibody (anti-HBc), surface antibody (anti-HBs), and anti-*Entamoeba histolytica* antibody (anti-Eh) are performed routinely for each patient on the first visit to our clinic. History of syphilis was defined as baseline positive TPHA and RPR titer <1:8. Exposure to hepatitis B virus (HBV) was defined as those either with positive HBsAg, anti-HBs, or anti-HBc [6], because in Japan, universal HBV vaccination is not conducted, except for health care professionals [9]. Patient visit our clinic at least every three months to receive renewal prescription of medications, since the prescription period under the Japanese health care system is limited to three months. Laboratory data, as well as baseline demographics (age, sex, ethnicity, treatment status for HIV infection, history of syphilis, history of AIDS, and history of HBV vaccination) on the first visit were collected from the medical records.

Social demographics variables were collected through a structured interview conducted by a clinical nurse specialist on the first visit as part of routine clinical practice [10]. The interview included the following behavioral variables: perceived route of transmission, sexuality (MSM status was based on self-identification), history and type of illicit drug used (intravenous injection or methamphetamine), gay’s bathhouse use, and health insurance status. Because the interview could underestimate the prevalence of illicit drug use, we also reviewed the medical records for information on illicit drug use and related variables covering the period from the first visit to December 2015.

Incident syphilis was defined as development of syphilis in the study patients. The diagnosis of syphilis was based on both serum RPR ≥8 and positive TPHA result [8], either with or without clinical symptoms/signs suggestive of syphilis. At our clinic, written informed consent is obtained from each patient to routinely store blood samples at the first and subsequent visits, thus we had the stored samples collected from each patient at least every three months [7]. Except for the patients who were clinically diagnosed of incident syphilis, the result of either the latest serum syphilis test conducted in clinical practice or the same test using the latest stored serum sample were used to determine the status of syphilis, whichever the follow-up time was longer. Censoring was performed for cases with incident syphilis, those referred to other hospitals, those who were lost to follow-up, or those who died, or at end of the observation period (December 31, 2015).

Incident syphilis was classified into early syphilis (including primary, secondary, and early latent syphilis), late syphilis (including late latent syphilis and syphilis with unknown duration), and others, including neurosyphilis and ocular syphilis [11,12]. We used the standard definitions for stages of syphilis; early latent being asymptomatic syphilis that was confirmed to be infected within a year from diagnosis, late latent being asymptomatic syphilis confirmed to be infected more than a year before diagnosis, and syphilis with unknown duration being asymptomatic syphilis that could not be classified into either early latent or late latent [11,12]. The date of development of incident syphilis was defined as the day of clinical diagnosis for patients with early syphilis. For patients who developed late or neurosyphilis or those diagnosed with syphilis based on stored serum samples, stored samples were backwardly tested for syphilis using samples collected every three months to determine the day of the first positive syphilis result, and thence the date of development of incident syphilis.

### Statistical analysis

The study patients were categorized into two groups; patients with incidence syphilis and those without syphilis. The incidence of syphilis was calculated by dividing the number of incident syphilis by person-time at risk. Person-time represented the time from the first visit to the date of incident syphilis as described above in patients who developed incident syphilis, and to the last negative syphilis test in patients without incident syphilis. Poisson regression was used to compare the incidence of syphilis among four observation periods (2008–2009, 2010–2011, 2012–2013, and 2014–2015).

Patients’ characteristics and social demographics were compared between patients with incident syphilis and those without such infection using the Student’s *t*-test for continuous variables and using either the χ^2^ test or Fisher’s exact test for categorical variables. The univariable Cox proportional hazards regression analysis was used to estimate the effect of each variable on the incidence of syphilis infection, and each variable with *p* value less than 0.1 was incorporated into multivariable analysis. Incarceration due to drugs and AIDS were not added to the multivariable model because of their multicollinearity with illicit drug use and CD4 count, respectively.

Statistical significance was defined at two-sided *p* values of <0.05. We used the hazard ratios (HRs) and 95% confidence intervals (95% CIs) to estimate the effect of each variable on the incidence of syphilis infection. All statistical analyses were performed with The Statistical Package for Social Sciences ver. 23.0 (SPSS, Chicago, IL).

## Results

**Fig 1:**
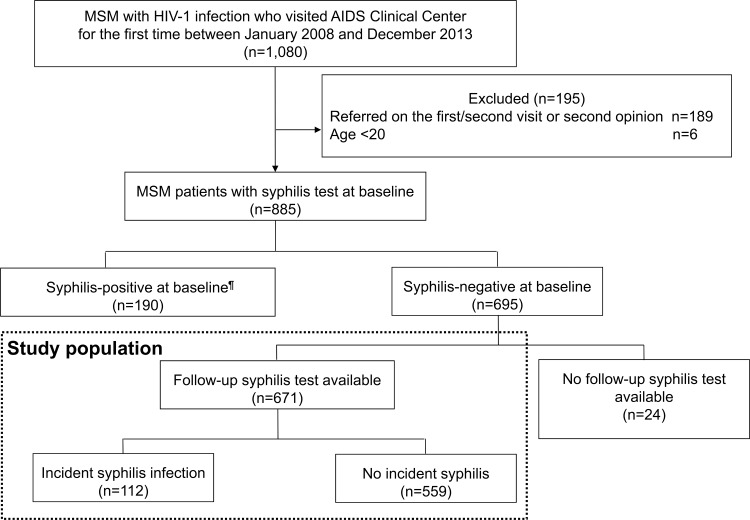
Patient enrollment process. ^¶^Syphilis was defined by Rapid Plasma Reagin ≥8 and positive *Treponema pallidum* latex agglutination. MSM: men who have sex with men.

A total of 1,080 MSM patients with HIV-1 infection visited our center during the study period, and 885 patients had a complete baseline data for analysis (Fig 1). Of these, 190 (21%) patients had active syphilis at baseline and were excluded. We also excluded another group of 24 patients due to lack of follow-up syphilis test, and thus data of 671 patients were analyzed as the study patients. The characteristics of the above 885 patients are summarized (S1 Table).

**Fig 2:**
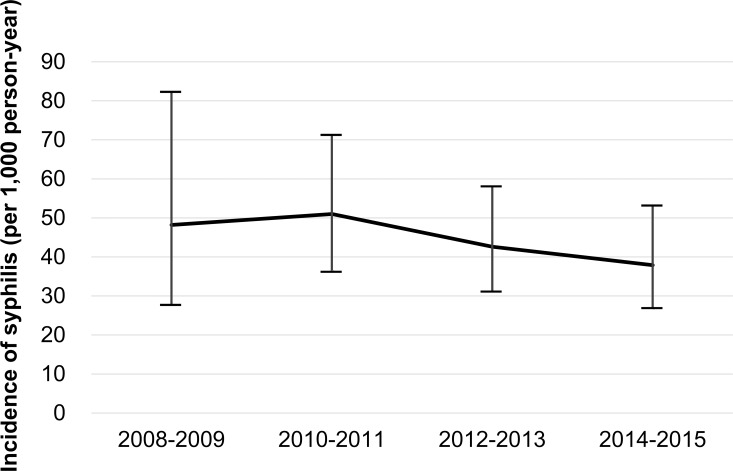
Incidence of syphilis among HIV-1-infected MSM during the observation period. The incidence decreased slightly during the observation period, although this trend was not significant (p = 0.315). Upper and lower whiskers represent 95% confidence interval.

In this study, 112 patients of 671 (17%) developed incident syphilis infection over 2,562 person-years of the total observation period, with an incidence rate of 43.7 per 1,000 person-year [95% CI, 36.5–52.3]. The median time from the first visit to the incident syphilis was 1.92 years (IQR 1.11–3.45, range 0.12–6.85). The incidence slightly decreased during the observation period, although the trend was not significant [2008–2009: 48.2 per 1,000 person-year (12 cases per 249 person-year), 2010–2011: 51.1 per 1,000 person-year (32 cases per 627 person-year), 2012–2013: 42.6 per 1,000 person-year (37 cases per 868 person-year), 2014–2015: 37.9 per 1,000 person-year (31 cases per 819 person-year), p = 0.315](Fig 2). With 885 patients who had complete baseline data as the denominator, 302 (34%) patients were/became syphilis-positive during the study period.

Table 1 summarized the characteristics of the study patients, including 112 who developed incident syphilis and 559 who did not. The study patients were mostly Japanese men of relatively young age (median: 36 years), covered with health insurance. Most patients were antiretroviral therapy (ART)-naïve at baseline, with a median baseline CD4 count of 238 /μl. Furthermore, 27% had history of syphilis and 58%, 9%, and 22% were exposed to HBV, were positive for HBsAg, and positive for anti-EhAb, respectively. The table also shows that 29% were illicit drug users and 46% visited gay’s bathhouse. Patients with incident syphilis were significantly younger (p<0.001), more likely to be exposed to HBV (p = 0.006), had history of syphilis infection (p<0.001) and positive for anti-EhAb (p = 0.003), and used illicit drugs (p = 0.022) than those without incident syphilis (Table 1). Furthermore, incident syphilis cases had marginally higher CD4 count (p = 0.054) and were less likely to have history of AIDS, albeit insignificantly (p = 0.091), compared with the no-syphilis group.

**Table 1:** Baseline characteristics of the study patients.

	All patients (n = 671)	Patients with incident syphilis (n = 112)	Patients without incident syphilis (n = 559)	P value
Age (years), median (IQR)	36 (30–43)	34 (27–38)	37 (31–44)	<0.001
CD4 count (/μl), median (IQR)	238 (85–374)	274 (152–404)	228 (77–372)	0.054
HIV-1 viral load (log_10_/ml), median (IQR)	4.77 (4.08–5.36)	4.79 (3.99–5.28)	4.77 (4.08–5.38)	0.750
On antiretroviral therapy, n (%)	65 (10)	11 (10)	54 (10)	1.000
History of AIDS, n (%)	202 (30)	26 (23)	176 (32)	0.091
History of syphilis, n (%)	183 (27)	53 (47)	130 (23)	<0.001
Anti-*Entamoeba histolytica* antibody-positive^¶^, n (%)	146 (22)	36 (32)	110 (20)	0.003
Hepatitis C RNA-positive, n (%)	3 (0.4)	1 (0.9)	2 (0.4)	0.422
Hepatitis C antibody-positive, n (%)	17 (3)	3 (3)	14 (3)	1.000
Hepatitis B surface antigen-positive, n (%)	61 (9)	12 (11)	49 (9)	0.476
Exposure to hepatitis B virus, n (%)^*^	388 (58)	78 (70)	310 (56)	0.006
Ethnicity, n (%)				0.370
Japanese	634 (95)	106 (95)	528 (94)	
Other Asians	22 (3)	2 (2)	20 (4)	
Others	15 (2)	4 (3)	11 (2)	
Illicit drug use, n (%)	195 (29)	43 (38)	152 (27)	0.022
Injection drug use, n (%)	51 (8)	5 (5)	46 (8)	0.239
Methamphetamine use, n (%)	44 (7)	10 (9)	34 (6)	0.294
Bathhouse use, n (%)	309 (46)	57 (51)	252 (45)	0.320
Incarceration due to illicit drugs, n (%)	19 (3)	6 (5)	13 (2)	0.109
Health insurance status, n (%)				0.485
With insurance	616 (92)	106 (95)	510 (91)	
No insurance	10 (1)	1 (1)	9 (2)	
On social benefits	45 (7)	5 (4)	40 (7)	
Follow-up period (years), median (IQR)	3.80 (2.19–5.66)	1.92 (1.11–3.45)	4.21 (2.50–5.87)	<0.001

History of syphilis was defined as baseline positive TPHA and RPR titer <1:8. Exposure to HBV was defined as those either with positive HBsAg, anti-HBs, or anti-HBc

^¶^ The variable anti-amoeba antibody was missing in 21 (3%) patients.

* Two patients were vaccinated with hepatitis B vaccine and counted as no exposure to hepatitis B virus.IQR: interquartile range,

The majority of incident syphilis comprised early symptomatic syphilis (n = 69, 62%), and the most common features were skin rash (n = 50, 45%), oral/pharyngeal lesions (n = 8, 7%), and genital lesions (n = 6, 5%) (Table 2). The group also included two patients with ocular syphilis. Asymptomatic syphilis was diagnosed in 41 (37%) of the 112 incident syphilis cases, including 4 (4%) early latent, 20 (18%) late latent, and 17 (15%) latent syphilis cases diagnosed by examination of stored serum samples.

**Table 2:** Stages and clinical features of incident syphilis (n = 112).

	Incident syphilis (n = 112)
Early symptomatic syphilis	69 (62%)
Skin rash	50 (45%)
Oral/pharyngeal lesions	8 (7%)
Genital lesions	6 (5%)
Lymphadenopathy	2 (2%)
Hepatotoxicity	1 (1%)
Symptom unknown	2 (2%)
Ocular syphilis	2 (2%)
Latent syphilis	41 (37%)
Early latent syphilis	4 (4%)
Late latent syphilis	20 (18%)
Latent syphilis diagnosed using stored serum samples	17 (15%)

Incidence of syphilis was particularly high among young patients (age <33 years: 60.1 per 1,000 person-year, 33–40 years: 54.2 per 1,000 person-year, >40 years: 18.8 per 1,000 person-year), those who were incarcerated due to illicit drug use (106.6 per 1,000 person-year), those with history of syphilis at baseline (86.6 per 1,000 person-year), and those with high baseline CD4 count (CD4 ≥350 /μL: 59.6 per 1,000 person-year, 200–349 /μL: 50.7 per 1,000 person-year, <200 /μL: 29.4 per 1,000 person-year) (Table 2). Univariate analysis showed a significant relationship between incident syphilis and younger age, (age <33 years versus age >40, HR 3.1, 95%CI 1.83–5.42, p<0.001; age 33–40 versus age >40, HR 2.9, 95%CI 1.66–4.94, p<0.001), history of syphilis infection (HR 2.8, 95% CI 1.96–4.13, p<0.001), HBV exposure (HR 1.6, 95% CI 1.07–2.39, p = 0.023), anti-Eh (HR 1.8, 95% CI 1.17–2.62, p = 0.006), higher baseline CD4 count (CD4 ≥350 /μL versus CD4 <200 /μL, HR 2.0, 95%CI 1.27–3.13, p = 0.003; CD4 200–349 /μL versus CD4 <200 /μL, HR 1.7, 95%CI 1.08–2.72, p = 0.023), and history of AIDS (AIDS versus no such history, HR 0.6, 95% CI 0.41–0.99, p = 0.044). Incident syphilis was also significantly associated with illicit drug use (HR 1.6, 95% CI 1.08–2.32, p = 0.018) and incarceration due to illicit drug use (HR 2.5, 95% CI 1.11–5.75, p = 0.028).

Multivariate analysis identified young age (age <33 years versus age >40, adjusted HR 4.0, 95%CI 2.22–7.18, p<0.001; age 33–40 versus age >40, adjusted HR 2.9, 95%CI 1.61–5.10, p<0.001), history of syphilis infection (adjusted HR 3.0, 95%CI 2.03–4.47, p<0.001), positive baseline anti-Eh (adjusted HR 1.8, 95%CI 1.17–2.68, p = 0.006), and higher baseline CD4 count (CD4 ≥350 /μL versus CD4 <200 /μL, HR 1.6, 95%CI 1.00–2.53, p = 0.050) as independent risk factors for incident syphilis (Table 3).

**Table 3:** Results of uni- and multi-variable analyses to estimate risk factors for incident syphilis infection.

	IR/1000 person-year	Univariable analysis (n = 671)			Multivarible analysis (n = 650)		
		HR	95% CI	P value	Adjusted HR	95% CI	P value
Age^¶^							
<33 years	60.1	3.1	1.83–5.42	<0.001	3.9	2.15–7.00	<0.001
33–40 years	54.2	2.9	1.66–4.94	<0.001	2.8	1.58–5.02	<0.001
>40 years	18.8	Ref	Ref		Ref	Ref	
History of syphilis at baseline vs. no history*	86.6 vs. 29.7	2.8	1.96–4.13	<0.001	3.0	2.03–4.47	<0.001
Baseline HBV exposure vs. -no exposure*	50.6 vs. 32.0	1.6	1.07–2.39	0.023	1.3	0.86–2.03	0.204
Baseline anti-Eh-positive vs. negative*	63.3 vs. 36.0	1.8	1.17–2.62	0.006	1.8	1.17–2.68	0.007
Baseline CD4 count (/μL)*		1.0	1.00–1.00	0.007	1.0	1.00–1.00	0.088
<200	29.4	Ref	Ref		Ref	Ref	
200–349	50.7	1.7	1.08–2.72	0.023	1.4	0.85–2.21	0.194
≥350	59.6	2.0	1.27–3.13	0.003	1.6	1.00–2.53	0.050
Baseline HIV-RNA load (per log_10_/ml increase)		0.9	0.81–1.10	0.467			
History of AIDS vs. no history	31.0 vs. 48.7	0.6	0.41–0.99	0.044			
Illicit drug users vs. non-users*	59.0 vs. 36.8	1.6	1.08–2.32	0.018	1.4	0.91–2.00	0.136
Injection drug users vs. non-users	25.2 vs. 44.4	0.6	0.23–1.39	0.212			
Methamphetamine users vs. non-users	67.5 vs. 41.5	1.6	0.84–3.07	0.156			
Incarceration due to drugs vs. no such history	106.6 vs. 41.6	2.5	1.11–5.75	0.028			
Bathhouse users vs. non users	46.6 vs. 40.9	1.0	0.73–1.48	0.815			

* These variables were incorporated into the model for multivariate analysis.

History of syphilis was defined as baseline positive TPHA and RPR titer <1:8. Exposure to HBV was defined as those either with positive HBsAg, anti-HBs, or anti-HBc

IR: incident rate, HR: hazard ratio, CI: confidence interval, HBV: hepatitis B virus, anti-Eh: anti-*Entamoeba histolytica* antibody

## Discussion

This present study investigated the incidence and risk factors of incident syphilis among HIV-infected MSM at a large urban HIV clinic in Tokyo, using clinical data and stored blood samples taken every three months for screening and determination of the date of incident syphilis. Syphilis is an endemic in this population in Japan; 34% of the patients were either infected with syphilis at baseline or infected with syphilis during the follow-up. Among HIV-infected MSM who were free of active syphilis infection at baseline, 112 patients (17%) developed incident syphilis infection, with an incidence of 43.7 per 1,000 person-year [95% CI, 36.5–52.3]. Younger age, history of syphilis infection at baseline, positive anti-Eh, and higher baseline CD4 count were identified as risk factors for incident syphilis. In particular, the incidence of syphilis among patients aged <33 years was more than three times higher than that among those aged >40 (60.1 versus 18.8 per 1,000 person-year). Interestingly, 37% of patients with incident syphilis were asymptomatic, supporting the importance of regular syphilis screening in HIV-1-infected MSM.

The present study has three important features. First, it is the first study that determined the prevalence and incidence of syphilis among HIV-1-infected MSM in Japan. The incidence of syphilis 43.7 per 1,000 person-year in HIV-1-infected MSM was substantially higher than the reported incidence of 1.0 per 100,000 population in Japan, or 1.6 per 100,000 male population in 2013 [5]. The American CDC guidelines recommend screening sexually active patients with HIV-1 infection for syphilis at least once annually, and every 3–6 months for those with multiple or anonymous partners [13]. The present study showed that this recommendation should be also strictly applied to HIV-1-infected MSM in Japan, especially young subjects with history of syphilis (based on positive TPHA with RPR titer <1:8 at first visit), and positive anti-Eh. As invasive amoebiasis, primarily a oral-fecal disease, is an emerging sexually transmitted disease in East Asia including Japan [14], positive anti-Eh can be one of the markers for unsafe sexual practice among HIV-1 infected MSM in this region.

Second, our study screened all subjects for syphilis, either clinically or using stored serum samples. A few studies reported the incidence of syphilis among HIV-1-infected MSM [15], however, most of them solely used surveillance data for syphilis or test results for syphilis from the central laboratory [16,17], and it is likely that they underestimated the incidence by overlooking latent syphilis. Importantly, our study used stored serum samples that were taken every three months to determine the date of incident syphilis for patients with late latent syphilis or syphilis of unknown duration, and hence succeeded in providing accurate estimation of incident syphilis. The incidence of syphilis in HIV-1-infected MSM is high, however, the study showed that the incidence did not increase during the observation period. Although we only focused HIV-1-infected MSM in this study, this finding seems to be in accordance with the surveillance data that showed the rapid increase in reported syphilis in recent years in Japan is largely due to increase in heterosexual transmission (2015: of the 2698 cases with syphilis, 585 had homosexual contact, 840 were heterosexual males, and 764 were heterosexual female [4], 2013: of the 1226 cases with syphilis, 443 had homosexual contact, 418 were heterosexual males, and 237 were heterosexual females [5]), which is different from other resource-rich countries where syphilis epidemic is mostly among MSM [2,18,19].

Third, detailed data for the clinical presentation of syphilis were collected in this study. The data showed that 37% of patients with incident syphilis were asymptomatic/latent, including 15% who were diagnosed by examining stored serum samples. The result is in agreement with that of previous reports [20,21] and highlights both the importance of regular screening among HIV-1-infected MSM and the difficulty of making diagnosis of syphilis in patients who do not regularly visit the health care facility.

Several limitations need to be acknowledged. First, due to the nature of single-center study, the results of this study might not necessarily represent the trend of syphilis infection among all HIV-1-infected MSM in Japan. However, as mentioned above that our clinic treats approximately 15% of the total HIV patients in Japan and because most HIV-1 infected MSM reside in urban areas such as Tokyo metropolitan area where most syphilis cases are also reported [3,4], any discrepancy between the study patients and HIV-1-infected MSM at large in Japan should be small. Second, because this study censored patients with incident syphilis during the observation period and did not take into account re-infection of syphilis of such patients, the actual incidence of syphilis among this population could probably be higher than the incidence of 43.7 per 1,000 person-year as reported in this study. This even highlighted the importance of regular syphilis screening among HIV-1-infected MSM. Third, it was difficult to avoid underreporting of illicit drug use and associated variables [22], and this might affect the results of association analysis between incident syphilis and drug-associated variables.

In conclusion, our study showed a constantly high incidence of syphilis in HIV-1-infected MSM (43.7 per 1,000 person-year) attending a large urban HIV clinic in Tokyo. Young age, history of syphilis infection, positive anti-Eh, and high baseline CD4 count were identified as risk factors for incident syphilis infection. Furthermore, 37% of patients with incident syphilis were asymptomatic. Regular syphilis screening and promotion of safe sex among HIV-1-infected MSM, especially young patients and those with a history of syphilis are urgently needed in order to lower the incident syphilis infection among this population.

## Supporting Information

S1 Table Characteristics of the patients with available baseline data (n = 885). (PDF)
