# Supplementary material for: Incidence and Risk Factors for Incident Syphilis among HIV-1-Infected Men Who Have Sex with Men in a Large Urban HIV Clinic in Tokyo, 2008−2015
Source: PLoS One. 2016 Dec 16;11(12):e0168642. doi: 10.1371/journal.pone.0168642 (PMC5161506; doi:10.1371/journal.pone.0168642)
Supplement: S1 Table — (PDF) [file pone.0168642.s001.pdf]

S1 Table1. Characteristics of the patients with available baseline data (n=885).

	All (n=885)	Study subjects: Syphilis negative at baseline with follow-up test* (n=671)	Excluded: Syphilis positive at baseline (n=190)	Excluded. No follow-up syphilis test (n=24)
Age (years), median (IQR)	36 (20-75)	36 (30-43)	36 (30-40)	38 (25-52)
CD4 count (/µl), median (IQR)	245 (89-374)	238 (85-374)	270 (95-386)	305 (89-361)
HIV-1 viral load ($\log_{10}$/ml), median (IQR)	4.77 (4.04-5.32)	4.77 (4.08-5.36)	4.76 (4.00-5.28)	4.69 (3.30-5.31)
On antiretroviral therapy, n (%)	92 (10)	65 (10)	23 (12)	4 (17)
AIDS, n (%)	266 (30)	202 (30)	55 (29)	9 (38)
TPHA-positive	376 (43)	183 (27)	190 (100)	4 (17)
Anti-*Entamoeba histolytica* antibody-positive*	199 (23)	146 (22)	50 (26)	3 (13)
Hepatitis C RNA-positive	3 (0.3)	3 (0.4)	0 (0)	0 (0)
Hepatitis C antibody-positive	21 (2)	17 (3)	3 (2)	1 (4)
Hepatitis B surface antigen-positive	76 (9)	61 (9)	13 (7)	2 (8)
Exposure to hepatitis B virus[¶]	539 (61)	388 (58)	134 (71)	17 (71)
Ethnicity, n (%)				
Japanese	839 (95)	634 (95)	182 (96)	23 (96)
Other Asians	26 (3)	22 (3)	4 (2)	0 (0)
Others	20 (2)	15 (2)	4 (2)	1 (4)
Illicit drug use, n (%)	266 (30)	195 (29)	64 (34)	7 (29)
Injection drug use, n (%)	67 (8)	51 (8)	15 (8)	1 (4)
Methamphetamine use, n (%)	60 (7)	44 (7)	15 (8)	1 (4)
Bathhouse use, n (%)	406 (46)	309 (46)	89 (47)	8 (33)
Incarceration due to illicit drugs, n (%)	27 (3)	19 (3)	8 (4)	0 (0)
Health insurance status, n (%)				
With insurance	797 (90)	616 (92)	160 (84)	21 (88)
No insurance	14 (2)	10 (1)	4 (2)	0 (0)
On social benefits	74 (8)	45 (7)	26 (14)	3 (13)

*The variable anti-amoeba antibody was missing in 32 (4%) patients.

[¶]Two patients were vaccinated with hepatitis B vaccine and counted as no exposure to hepatitis B virus.

IQR: interquartile range, TPHA: treponema pallidum latex agglutination.
